# Validation and application of a visual LAMP assay for Mpox diagnosis — Insights from clinical samples in Thailand^☆^

**DOI:** 10.1016/j.mex.2025.103568

**Published:** 2025-08-15

**Authors:** Wansadaj Jaroenram, Yada Ongchanchai, Ravee Nitiyanontakij, Pawita Suwanwatthana, Ratchana Ponang, Wansika Kiatpathomchai

**Affiliations:** a National Center for Genetic Engineering and Biotechnology (BIOTEC), 113 Thailand Science Park, Phahonyothin Rd., Klong Neung, Klong Luang, Pathum Thani 1212, Thailand; b Faculty of Veterinary Technology, Kasetsart University, Bangkok 10900, Thailand; c Bamrasnaradura Infectious Diseases Institute, 126 Tivanon Rd., Talat Khwan, Mueang, Nonthaburi 11000, Thailand

**Keywords:** Colorimetric LAMP, Monkeypox virus, Mpox, Xylenol orange, XO

## Abstract

Despite numerous recent advances in infectious disease diagnostic technologies including quantitative (q) polymerase chain reaction (PCR) and antigen test kit (ATK), there remains a need to develop more efficient point-of-care assays for large-scale operations. In an effort towards such a screening test for low-resource settings, we have established a colorimetric loop-mediated isothermal amplification assay and detected the amplified DNA via naked-eye observation to maximize assay simplicity using monkeypox (Mpox) as a model. The assay is cheap, highly sensitive and specific to Mpox with the potential for translation into broader applications.

• Our protocol offers a simpler, faster, and cheaper test for Mpox than the current gold-standard qPCR, with 100% accuracy.
• assay enables decentralized screening and provides visually interpretable results immediately after incubation
• The versatility of this protocol allows for easy adaptation to detecting new or re-emerging diseases, utilizing a visual readout for maximum simplicity.

## Highlights

• Our protocol offers a simpler, faster, and cheaper test for Mpox than the current gold-standard qPCR, with 100 % accuracy.
• The assay enables decentralized screening and provides visually interpretable results immediately after incubation.
• The versatility of this protocol allows for easy adaptation to detecting new or re-emerging diseases, utilizing a visual readout for maximum simplicity.

## Graphical abstract

**Figure:**
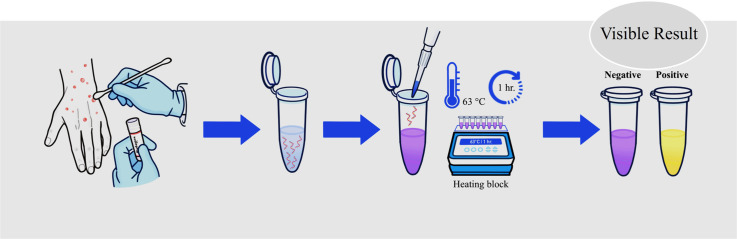

**Specifications table**

**Table:** 

**Subject area**	Biochemistry, Genetics and Molecular Biology
**More specific subject area**	Molecular Biology
**Name of your protocol**	OX-based cLAMP for Mpox detection
**Reagents/tools**	**Reagents** • *Bst* 2.0 WarmStart® DNA Polymerase (New England Biolabs, Cat. no M0538; 8000 U mL^-1^) • dNTPs (Thermo Fisher Scientific™, Cat. no R0191) • UltraPure™ DNase/RNase-Free Distilled Water, molecular grade (Thermo Scientific™, Cat. no 10,977–015) • Xylenol orange disodium salt (Merck Millipore, Cat. no 52,097) • Primers (Reagent Setup) • p-GEM® - T Easy Vector System (Promega, Cat no A1360) • GeneJET™ Plasmid Miniprep Kit (Thermo Scientific™, Cat no K0503) • Ampicillin (Bio Basic, Cat. no AB0028) • Bacto™ Tryptone (BD Biosciences, Cat no 211,705) • Bacto™ Yeast Extract (BD Biosciences, Cat no 212,750) • Sodium chloride, for molecular biology (Merck Millipore, Cat. no S3014) • Agar, Technical (Solidifying Agent) (BD Diagnostic Systems Europe, Cat no 281,230) • Isopropyl β-d-1-thiogalactopyranoside (IPTG) (Merck Millipore, Cat. no I6758) • 5-Bromo-4-chloro-3-indolyl β-d-galactopyranoside (X-Gal) (Merck Millipore, Cat. no 10,651,745,001) • NucleoSpin® Gel and PCR Clean-up (Macherey-Nagel, Cat No 740,609.50) • Gel Loading Dye, Purple (6X), no SDS (New England Biolabs, Cat. no B7025S) • 2-Log DNA Ladder (0.1–10.0 kb) (New England Biolabs, Cat. no N3200) • DNase-free, RNase-free barrier pipette tips • Agarose, for molecular biology (Thermo Scientific™, Cat. no 75,510–019) • SERVA DNA stain G (SERVA Electrophoresis GmbH, Cat. no 39,803.01) • Ammonium sulfate ((NH_4_)_2_SO_4_) (Merck Millipore, Cat. no A4418) • Potassium chloride (KCl) (Bio Basic, Cat. no PB0440) • Magnesium sulfate (MgSO_4_) (Merck Millipore, Cat. no M7506-500G) • TWEEN® 20 Vetec™ reagent grade, 40 % (Merck Millipore, Cat. no V900548-SPEC-1L) • Potassium hydroxide (KOH) (EMSURE®, Cat. no 1.05033.1000) • Trizma® base, for molecular biology (Merck Millipore, Cat. no 93,362) • Acetic Acid, glacial (Merck Millipore., Cat. no A6283) • Ethylenediaminetetraacetic acid disodium salt (EDTA) (Merck Millipore, Cat. no E6511) • Viral Transport Medium (BioTrend, Germany, Cat. no VTM-1000ML) • *E coli.* strain JM109 competent cells (Promega, Cat. no L2005) • MagDEADx SV kit (Precision System Science Co., Ltd., Chiba, Japan) **Equipment** • Mini Heating Dry Bath Incubator (Major science, USA) • Veriti™ 96-Well Thermal Cycler (Applied Biosystems™, USA) • NanoDrop® ND-1000 Spectrophotometer (NanoDrop Technologies, Inc., USA) • Vortex Mixer Model: Genie 2 G560E (Scientific Industries, USA) • Loopamp Realtime Turbidimeter (LA-500) (Eiken Chemical Co., Japan) • Heraeus Pico Microcentrifuges (Thermo Scientific™, USA) • Mini-6KS Mini-Centrifuge (Allsheng, China) • Thermo Scientific ST8R Refrigerated Benchtop Centrifuge (Thermo Scientific™, USA) • Peltier-cooled incubator IPP55 (Memmert, Germany) • Incubator shaker KS 3000i control (IKA®, Germany) • Major Science MS 500V Programmable Power Supply for Electrophoresis (Major science, USA) • Mini Pro 300V Power Supply, MINI-300 (Major science, USA) • Syngene GeneGenius Bio Imaging System (Syngene, USA) • Mars Biological safety cabinets class 2 - Scanlaf (Labogene, Denmark) • Thermo Scientific Lab-Line "AquaBath" Water Bath (Thermo Scientific™, USA) • V.Go Magnetic Hot plate Stirrer (Chemoscience, Malaysia) • magLEAD® 12gC Automated extraction machine (Precision System Science Co., Ltd., Chiba, Japan) **Software**Bioinformatic software for the design of LAMP primers. • NEB® LAMP Primer Desing Tool Version 1.4.2. (https://lamp.neb.com/#!/). • All designed primers were validated for cross dimerization by using Net Primer Design (Premier Biosoft) and Basic local alignment search tool (BLAST) (https://blast.ncbi.nlm.nih.gov/Blast.cgi).
**Experimental design**	A xylenol orange-driven colorimetric LAMP diagnostic tool was developed to detect DNA using Mpox as a model. The tool has four modes of analyses: naked-eye observation using in-house pH-dependent indicator to maximize the visual detection and assay simplicity, UV–Vis spectrophotometry, gel electrophoresis and real-time turbidity measurement run on a Loopamp Realtime Turbidimeter (LA-500).
**Trial registration**	*N/A*
**Ethics**	This project received approval from the Institutional Review Board of Bamrasnaradura Infectious Diseases Institute with Ethics Committee document No S04 1h/66_ExPD.
**Value of the Protocol**	• Designed for low-resource environments, this protocol eliminates the need for specialized equipment and technical expertise, promoting accessibility and ease of use. • Its rapid, colorimetric readout enables screening at the point-of-care, minimizing turnaround time and facilitating outbreak containment. • The modular nature of the assay permits easy customization for emerging pathogens, enhancing preparedness in response-driven diagnostic settings.

## Background

Colorimetric loop-mediated isothermal DNA amplification (cLAMP) has emerged as a powerful diagnostic tool for infectious diseases, offering high sensitivity, specificity, and a simple visual readout—an ideal solution for low-resource settings [[1], [2], [3], [4]]. Despite these advantages, challenges such as limited scalability for high-volume testing [1,[3], [4], [5]], and poor color contrast between weak positives and negatives [3,[6], [7], [8], [9]] remain. To address these limitations, we developed a pH-dependent, xylenol orange (XO)-based cLAMP protocol, enhancing visual detection and diagnostic reliability. This approach builds on previous work in *Escherichia coli* detection [4] and has since been adapted for scale drop disease virus (SDDV) in Asian sea bass [5], cephalopod species identification [10], and *Vibrio parahaemolyticus* detection in raw seafood [11]. In this study, we further proved the validity of our cLAMP assay by using monkeypox virus (Mpox) as a model.

Briefly, Mpox, caused by a member of the Orthopoxvirus genus, is transmitted through close contact with infected individuals or contaminated materials [12]. As the 2022 global outbreak intensified, the World Health Organization (WHO) declared Mpox a public health emergency in August 2024, prompting increased efforts in surveillance, vaccination, and accessible testing [12]. Against this backdrop, our assay serves as a rapid, point-of-care diagnostic tool, designed to be adaptable for detecting other pathogens, thereby reinforcing preparedness for future emerging diseases [13].

Our Mpox-cLAMP-XO assay operates on a straightforward yet powerful principle: strand-displacement activity of *Bst* polymerase generates pyrophosphate and proton (*H*+) by-products [14,15] causing a pH drop in weak buffering conditions. This acidification triggers a distinct color shift in the XO dye, making the test easy to interpret visually. While several pH-sensitive dyes have been explored for LAMP reactions—including phenol red [16,17], leuco crystal violet [18,19], hydroxynaphthol blue (HNB) [[20], [21], [22]], calcein [[23], [24], [25], [26]] and malachite green (MG) [27]—many suffer from ambiguous transitions, particularly in weakly positive samples. For instance, calcein and HNB present indistinct color changes, while calcein's manganese ion requirement inhibits polymerase activity [23] and consequently decreases detection sensitivity [28,29]. Malachite green (MG) though offering an additional dark blue-to-clear transition, requires prolonged incubation (∼60 min) and exhibits moderate sensitivity (>100–1000 copies of target) [3,20,23,[30], [31], [32]]. Other dyes, such as bromocresol purple, bromothymol blue, neutral red, and cresol phthalein, face commercial restrictions due to intellectual property rights [33].

**Fig. 1:**
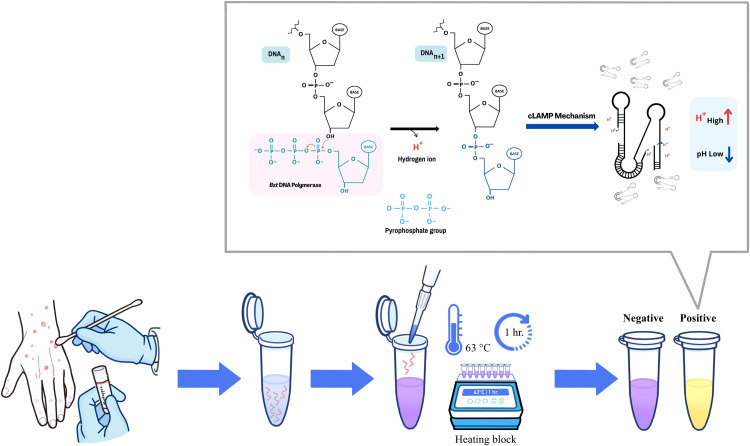
Diagnostic principle of the colorimetric Mpox-LAMP-XO assay. The entire assay consists of 3 steps as follows: Sample Preparation – DNA can be extracted using any suitable reagent. The assay is compatible with lesion surface swabs, or exudates. LAMP Incubation – The extracted DNA is added to the LAMP reaction mixture and incubated at 63 °C for 1 hour. This step can be performed using a heating block, thermal cycler, incubator, or real-time Turbidimeter. Result Interpretation – The outcome is determined by visual inspection. If the target DNA (F3L gene) is present, the reaction generates excess protons (*H*+), leading to a pH drop that changes the color of Xylenol Orange (XO) from purple to yellow. In the absence of the target, the solution retains its original purple hue. For addition readout formats i.e. gel electrophoresis, UV–Vis scanning method, and real-time turbidity measurement, please refer to the main text. The entire process from sampling to readout takes under 75 min and requires minimal hands-on time.

In contrast, XO (yellow < pH 6.7 < purple) offers superior color differentiation between positive and negative reactions. In the absence of target DNA of Mpox, the reaction remains purple, confirming a negative result. Conversely, the presence of target DNA leads to the accumulation of LAMP amplicons, which acidifies the solution and induces a distinct color shift from purple to yellow, significantly enhancing visual interpretation. The diagnostic principle of our Mpox-LAMP-XO assay is illustrated in Fig. 1.

## Description of protocol

### Material preparation

#### Primer design

LAMP primers were designed based on the F3L gene of the Mpox genome (GenBank No AF380138), which encodes the F3 protein believed to play a role in viral interactions with the host immune system [34,35]. The primer set includes outer primers (F3, B3), inner primers (FIP, BIP), and loop primers (LF, LB). All primers were designed using NEB® LAMP Primer Design Tool (Version 1.4.2) (https://lamp.neb.com/#!/) and computationally verified for cross-dimerization using Net Primer Design (Premier Biosoft) and BLAST (https://blast.ncbi.nlm.nih.gov/Blast.cgi).FIP and BIP primers, synthesized by linking two adjacent primers (40–45 bp), differ from the remaining primers, which are ∼20 nt in length with a GC content of 60 % (Tm ∼60 °C). Primer sequences are listed in Table 1.

**Table 1:** Primer sequences for Mpox DNA detection.

Primer name	Sequence (5′−3′) on F3L gene
F3	AGGCATAAAATGTAGGAGAGTT
B3	AGATTCTGATGCTGATGCTAT
FIP	ACCGTTATTAATGAGTACTGCCAAA—TTTT—GCCCCACTGATTCAATACG
BIP	ACCGGAATAACATCATCAAAAGACT—TTTT—ATAGATGATGTATCCCGCG
LF	CTAGGAGAGATTGGTCTTT
LB	ATTATCCTCTCTCATTGAT

#### Assay controls

Colorimetric LAMP provides qualitative results, indirectly indicating the presence of LAMP products. Contamination can lead to false positives, necessitating stringent controls. To mitigate this risk, two negative controls should be included at premix preparation and template addition steps to pinpoint contamination sources. If false positives occur, they can help locate the issue within the workflow. When using a heating block instead of a thermocycler, a positive control (e.g., plasmid DNA or genomic viral DNA) ensures the heat source remains effective. Laboratories adopting this protocol for other viral targets should conduct specificity tests against respiratory viruses and common hospital-acquired flora.

### Biological samples

Sample input should be purified, and the total nucleic acid must be eluted in nuclease-free water for the best results. Do not elute in TE buffer as it may inhibit the color development of the assay. Acidic samples may spontaneously turn the colorimetric LAMP reaction reddish-pink or yellow upon addition. Samples should not contain SDS as it is a strong inhibitor of amplification. If samples contain a trace of guanidine, ensure that the final concentration of guanidine remains <20 mM to avoid inhibiting the LAMP reaction [9].

### Reagent preparation

#### DNA primer stock

Primers, including outer primers (F3, B3), inner primers (FIP, BIP), and loop primers (LF, LB), are typically supplied as lyophilized pellets and should be stored at −20 °C upon arrival. To prepare a 100 μM stock, the pellets must be reconstituted in DNase-RNase-free water, following the manufacturer’s recommended volume. Then, the primer solutions should be vortexed and kept at 4 °C overnight to ensure complete dissolution of any residual material. The outer primers require further dilution in DNase-RNase-free water using a 1:9 volumetric ratio to create a 10 μM working stock, which should be stored at −20 °C until used.

#### Low-buffer stock for cLAMP reaction

Low-buffer for cLAMP reactions can be prepared in bulk at a desired volume and stored at room temperature in the dark vial until use. For a 10-mL 10 × low-buffer stock, mix 2 mL of 500 mM (NH_4_)2SO_4_, 5 mL of 1000 mM KCl, 2 mL of 100 mM MgSO_4_, and 0.1 mL of 100 % (v/v) Tween® 20. Adjust the pH to 8.5 using 1 M KOH, then bring the total volume to 10 mL with deionized water. The final 10 × stock contains 100 mM (NH_4_)_2_SO_4_, 500 mM KCl, 20 mM MgSO_4_, and 1 % (v/v) Tween-20. All components must be autoclaved prior to use, except Tween® 20, which is sensitive to heat and light.

#### TAE buffer stock

To prepare 1 L of 50 × TAE buffer, dissolve 242 g Trizma® base in 700 mL of deionized water. Combine 57.1 mL of glacial acetic acid and 100 mL of 0.5 M EDTA (pH 8.0) into the solution. Then adjust the final volume to 1 L total with deionized water. Autoclave the buffer before use.

#### Luria-Bertani (LB) broth medium

To prepare Luria-Bertani (LB) broth, dissolve 10 g of tryptone, 5 g of yeast extract, and 10 g of sodium chloride (NaCl) in approximately 800 mL of distilled water, ensuring all components are fully dissolved. Adjust the volume to 1 L with additional distilled water. If necessary, modify the pH to 7.0 ± 0.2 using NaOH or HCl. Sterilize the solution by autoclaving at 121 °C for 20–30 min on the liquid cycle. Allow the broth to cool to room temperature before use.

#### 5mM **xylenol orange (XO) working solution**

To prepare a 5-mM XO working solution, dissolve XO disodium salt (Millipore Sigma, MW 716.62 g/mol) 0.3583 g in water to make a final volume of 100 mL. Mix well by pipetting then sterilize by filtration through a 0.22-μm sterile filter. Keep the filtrate in a dark place at between 4 °C and room temperature until use.

## Protocol details

This protocol describes the development, testing, and validation of the Mpox-cLAMP assay in five key steps: genomic DNA extraction, recombinant plasmid preparation for assay optimization, and result analysis via four detection methods—naked-eye observation, UV–Vis spectrophotometry, gel electrophoresis, and real-time turbidity measurement. The following section outlines each step in detail

### **Monkeypox DNA extraction (∼ 30min)**

**Critical:** Samples must be eluted in DNase-RNase-free water. Any other non-low buffer solution, e.g., TE with/without SDS, will interfere with the color development of the cLAMP assay [9].

1. Collect nasopharyngeal or skin lesion swab samples from patients and store initially in viral transport medium (VTM).
2. Extract DNA from 150 μL of the original VTM stock by using the automated extraction machine (magLEAD® 12gC) with the MagDEADx SV kit (Precision System Science Co., Ltd., Chiba, Japan). Please refer to the guidelines and precautions specified in the user manual.
3. Reconstitute DNA in 50 μL DNase-RNase-free water and analyze by spectrophotometry to determine the quality and quantity of the extracted total DNA (optional). Store the DNA at −20 °C until use.

### Recombinant plasmid DNA preparation (∼ 3 days)

**Critical:** Preparation of plasmid DNA can span multiple days due to overnight bacterial culture. Plan this step carefully before starting. Steps 4–7 are performed according to the manufacturer’s protocol for pGEM®-T Easy Vector Systems.

4. Amplify a desired target region on the F3L gene of MPox by PCR using the forward and reverse primers (F3 and B3) listed in Table 1. (Note that F3L gene is used in this protocol as a model target to demonstrate the feasibility of the assay.) Program the thermocycler according to the following setting: 94 °C for 2 min (initial denaturation), then 30 cycles of 94 °C denaturation (30 s), 60 °C annealing (45 s) and 75 °C extension (45 s). The final extension was carried out at 75 °C for an additional of 2 min. Confirm the presence of specific PCR product of 221 bp in size by agarose gel electrophoresis (Steps 23–29).
Adjustment point: Lower or higher annealing temperatures may be used when optimizing conditions for different target sequences.
5. Ligate the PCR product with the pGEM®-T Easy Vector at EcoRI-HF™ cut sites. Confirm successful ligation by agarose gel electrophoresis. Transform the ligated pGEM®-T Easy Vector into *E. coli* (*E. coli*) strain JM109 competent cells by heat shock transformation at 42 °C, followed by incubation on ice for 2 min. Add 900 μL LB medium into the cells and incubate at 37 °C with shaking at 150 rpm for 90 min.
6. Spread the cells on IPTG/X-Gal plates and incubate at 37 °C for 16 hrs.
7. Pick the white transformants from the plates with clean pipet tips and inoculate them in LB medium containing 100 μg/mL ampicillin. Incubate the culture at 37 °C with shaking at 250 rpm for 16 hrs. Perform colony PCR on the selected transformants using the same set of forward and reverse primers to confirm a successful insertion of the PCR product into the pGEM®-T Easy Vector (See Troubleshooting, Table 3)
8. Extract the plasmids by using GeneJet Plasmid Miniprep kit.
9. Determine the concentration of the yielded plasmids by spectrophotometric analysis at 260/280 nm. Confirm the sequence by DNA sequencing.
10. Serially dilute the original stock of DNA by 10 folds in DNase-, RNase-free water to prepare the working template stocks, e.g., 50,000, 5000, 500, 50, 5 and 0.5 copies/μL. Store the plasmid template at −20 °C until use.

### **Colorimetric LAMP (∼ 60min)**

**Note:** This assay has been optimized for its detection target [36]. Any modification to the target sequence requires re-evaluating all reaction components, including temperature and time, using the quantities or concentrations listed in Table S1.

**Critical:** To minimize contamination, laboratories implementing this protocol should designate separate areas for premix preparation and template addition. Two sets of negative controls should be included at both stages to pinpoint potential contamination sources

11. Prepare a 23 μL premix solution by combining the corresponding specified volume of individual components to achieve the final concentration listed in Table S1.
12. Aliquot the 23 μL premix solution into individual PCR tubes.
13. Add 2 μL negative control into the tube assigned for pre-mix negative control. Mix well and close the cap.
14. In a space designated for template addition, add 2 μL negative control into the tube assigned for template-room negative control. Mix well and close the cap, add 2 μL of DNA template (total DNA or plasmid DNA) into the PCR tubes assigned for testing. Mix well by pipetting and closing the caps. Lastly, add 2 μL positive control into the tube assigned for positive control. Mix well and close the cap (See Troubleshooting, Table 3).
15. Pulse centrifuge the PCR tubes.
16. Place them in the heat source e.g. a standard thermal cycler, a heating block, or a real-time turbidimeter (for real-time analysis; see Step 30 - 31) set at 63 °C, and incubate for 60 min. If a heating block is used, it must be operated under a 100 - 105 °C-heated lid control to prevent evaporation of the LAMP solution.

### **Visual analysis by the naked-eye observation (endpoint analysis) (∼ 1min)**

**Fig. 2:**
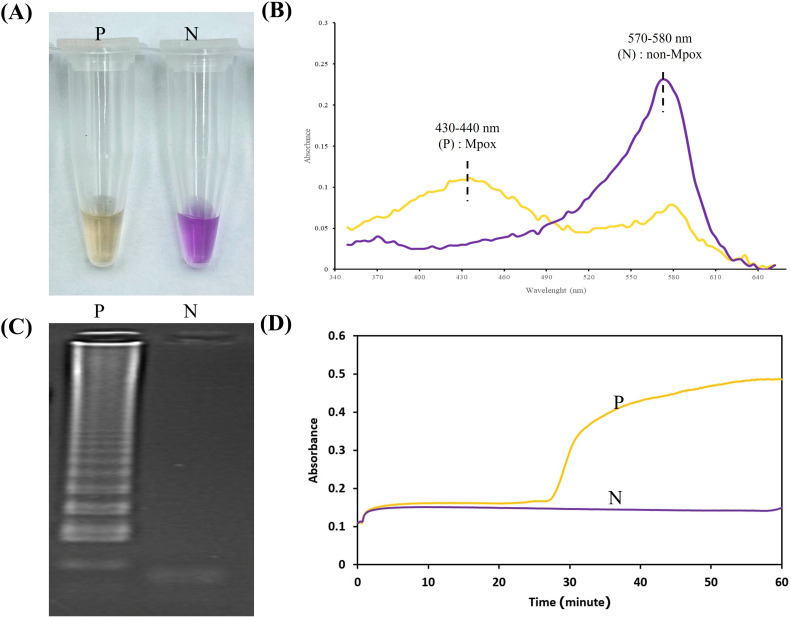
Guideline for the result analysis of Mpox-cLAMP. (A) Side-by-side comparison of positive (yellow) and negative (purple) outcomes. (B) UV–Vis analysis of positive (P, Mpox DNA), and negative (N, template-free reaction) results characterized by the absence of absorption peak at 430 nm in the negative sample. (C) Corresponding gel electrophoresis confirmation of colorimetric results shown in A. (D) Corresponding real-time turbidity measurement: a rising curve corresponds to Mpox-positive samples, while a flat or negligible curve indicates Mpox-negative results. P, N: positive control (1000 copies of plasmid), negative control (template-free reaction), respectively.

17. Once the run is finished, take the reaction tubes out of the heat source, and set them at room temperature for an additional 1 min to allow the color to fully develop.
18. Observe the final reaction colors and analyze the end results based on the guidelines shown in Fig. 2A. (See Troubleshooting, Table 3).

### **Optional analysis with UV–Vis spectrophotometry (endpoint analysis) (∼ 10min)**

**Note:** UV–Vis analysis is optional and serves as an objective method to minimize interpersonal bias in color interpretation across different observers. Researchers implementing this protocol on a customized automated platform may modify the UV–Vis measurement described in steps 19–21 to accommodate automated analysis.

19. Switch on the NanoDrop® Spectrophotometer and select the UV–Vis analysis option (300–700 nm) from the test menu. Perform a blank measurement using DNase-/RNase-free water.
20. Carefully pipette 2 μL of the final LAMP product onto the pedestal, ensuring the liquid forms a dome shape on the optical sensor. Lower the sampling arm gently until it contacts the pedestal, then initiate the analysis (See Troubleshooting, Table 3).
21. After analysis, lift the pedestal and use a Kimwipe to remove the residual sample before loading the next one. Avoid direct contact with the optical surface.
22. Examine the absorption spectra of Mpox-cLAMP following the guideline in Fig. 2B

### **Gel electrophoresis (endpoint analysis) (∼ 60min)**

**Critical:** During assay optimization, it is always recommended to analyze cLAMP products with agarose gel electrophoresis to confirm the presence of specific cLAMP products characterized by a ladder-like pattern on electropherogram.

23. Store cLAMP product at −20 to 4 °C for long term storage or analyze it immediately after visual analysis.
24. Prepare 2 % (w/v) agarose gel in 1 × TAE buffer. For instance, dissolve 2 g agarose in 100 mL 1 × TAE buffer in a microwave. Pour the fully dissolved agarose gel in the cassette already assembled with an appropriate comb size. Wait until the gel is firm and remove the comb.
**Critical:** Always add TAE slightly more than the theoretically required volume to compensate for evaporation during agarose gel preparation.
25. Prepare the electrophoretic chamber by placing the agarose gel in the chamber such that the comb side is facing the anode (-). Add 1 × TAE buffer into the chamber to cover the gel.
26. Mix 5 μL of individual cLAMP products with 1 μL 6 × DNA loading buffer thoroughly and load the combined mixture into the well.
Run the gel at 100–120 V for 30 min until the yellow dye front reaches the gel’s bottom edge.
28. Stain the gel in 0.1 % SERVA G solution in distilled water for 5 min, followed by de-staining in distilled water for another 10 min.
29. Visualize the LAMP products under UV light and interpret the result according to the guideline in Fig. 2C.

**Turbidity measurement (real time analysis) (real-time continuously during incubation)**

**Note:** This mode of analysis is applicable only when running LAMP reactions in the Loopamp Realtime Turbidimeter (LA-500) (Eiken Chemical Co., Japan). If the reactions are run in any standard thermal cycler or heating block, only endpoint modes can be used.

30. On incubating the cLAMP reactions in the LA-500, the device automatically measures turbidity every six seconds by detecting the by-product of the LAMP reaction (magnesium pyrophosphate), which causes a white precipitate. The instrument will display a real-time amplification curve on its screen
31. To interpret results: positive samples will show an increase in turbidity over time, represented by a rising curve. In contrast, negative samples will have a flat or negligible curve, indicating no amplification.
32. To record the data: use the built-in printer to obtain a hard copy of the results, or manually record the data displayed on the screen and then interpret the result according to the guideline in Fig. 2D
33. For post-reaction handling: once the reaction is complete, the LA-500 will heat the reaction blocks to inactivate the enzyme and stop the reaction. Safely dispose of the used reaction tubes following laboratory protocols.

## Protocol validation

### Detection limit validation

Our one-step cLAMP assay for Mpox DNA detection offers a versatility of analysis that can be accomplished visually, optically, and in real-time based on the equipment available at the site of implementation. In the most convenient and affordable mode, the test outcomes can be clearly observed with the naked-eye through the change of reaction hue from purple to yellow. This instrument-free colorimetric analysis lends our techniques readily exploitable for mass screening applications that can be performed in decentralized settings. While the uniqueness of our methods is attributed to colorimetry, the LAMP assays described in this protocol are also compatible with conventional agarose gel electrophoresis to confirm the presence of LAMP products. Additionally, the change in colorimetry can be quantitatively measured by UV–Vis spectrophotometry because each observable reaction hue has a specific optical fingerprint in its absorption peaks. Therefore, it is feasible to further develop an optical reader for instantaneous determination of the test results while the samples are being incubated to accommodate a large-scale, high-throughput testing in the future. The prospect of having a fully integrated automated system to read the test outcomes will also minimize interpersonal bias in the process of result determination that involves a large volume of samples. Lastly, the assay result can be measured in a real-time manner. This reading mode enables quantitative analysis by correlating the time to amplification with the initial template concentration.

**Fig. 3:**
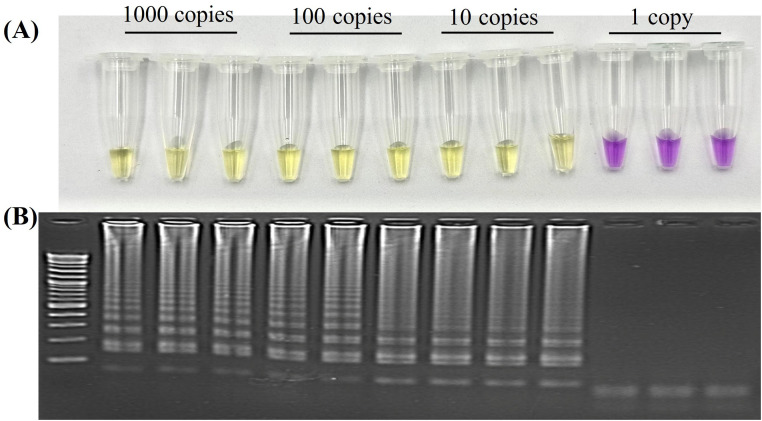
Sensitivity of Mpox-cLAMP by plasmid. (A) Visual detection limit demonstrated at 10 plasmid copies of the F3L target gene. (B) Corresponding gel electrophoresis confirmation of colorimetric results shown in A.

The testing steps and the optimal concentration of our assay component, experimentally determined in-house, was listed on Table S1. Clearly, our assay is highly sensitive, and able to achieve a detection limit (LoD) of 10 viral genomic copies per reaction when using plasmid DNA as a template (Fig. 3A and 3B). This LoD is 2 times and 10 times greater than the respective real-time LAMP protocols described by Feng et al. (2022) [37] and Lizuka et al. (2009) [38].

### Specificity validation

**Fig. 4:**
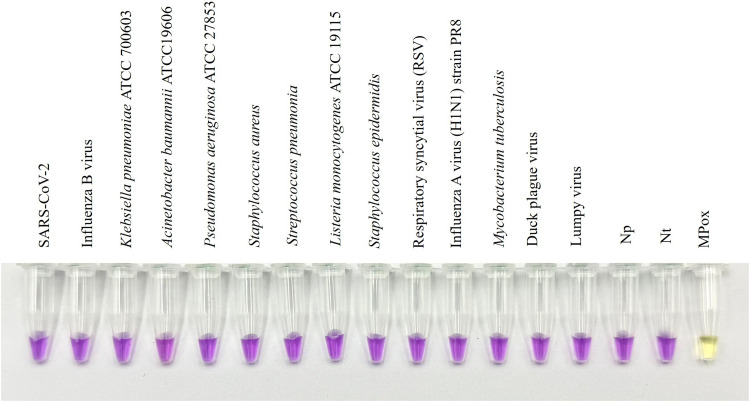
Molecular specificity of Mpox-cLAMP. The assay exhibited no cross-reactivity with clinically infectious pathogens, including important respiratory viruses (e.g. SARS-CoV-2 and Influenza A and B), other Poxviridae members (e.g. Lumpy virus and Duck plague virus), and common hospital-acquired bacteria e.g. P.aeruginosa and S. aureus. Np and Nt: template-free negative control prepared in the premix room and template-free negative control prepared in template addition room.

When tested against a panel of other related pathogens, our assay exhibits no cross-reactivity with these viruses based on the color change. This result corresponded with that of AGE, reinforcing the fidelity of our detection target that is exclusively specific to Mpox (Fig. 4). It is worth noting that cowpox is closely related to mpox and would ideally be included in specificity testing. Although we were unable to obtain cowpox DNA, we performed in silico analyses to assess potential cross-reactivity. Genome alignment showed 97.28 % identity and 89 % query coverage with Mpox (Fig. S1, A). However, primer alignment revealed no significant cross-binding with the cowpox genome (Fig. S1, B), indicating low cross-reactivity risk. Nonetheless, we acknowledge that experimental validation remains essential for definitive specificity determination. We plan to incorporate cowpox testing into future work once the sample becomes available.

### Clinical validation

To provide a complete picture of the assay’s performance in the clinical testing context, we demonstrated the feasibility of our assays in a proof-of-concept testing using 100 total DNA samples isolated from patient’s nasopharyngeal swabs/blood/skin lesion swabs. We correctly identified all 50 negative samples and 50 positive samples whose positive Ct (cycle threshold) values range from 16.5 to 32 (lowest from our archive samples) according to the standard qPCR assay (BioPerfectus Technologies, Taizhou, China) (Fig. S2, sample ID 41–90). The statistical sensitivity, specificity, and accuracy were 100 % (Table 2). The origin (sample type) of each sample was indicated in Table S2.

**Table 2:** Summary of clinical validation.

Colorimetric LAMP	Mpox status by reference assays	
	Positive	Negative
Positive	50 (TP)^a^	0 (FN)
Negative	0 (FP)	50 (TN)
% Concordance with respect to qPCR results	100 (sensitivity)	100 (specificity)
	100 (accuracy)	

Sensitivity = [TP/(TP+FN)] × 100, Specificity = [TN/(TN+FP)] × 100.

Accuracy = [(TP+TN)/(TP+TN+FN+FP)] × 100.

^a^TP, true positive; FP, false positive; FN, false negative; TN, true negative.

**Table 3:** Recommended solutions to the potential problems in the protocol.

**Step**	**Problem**	**Possible Cause(s)**	**Solution**
**Reagent Setup –** Low-buffer	Cloudiness or precipitation in the isothermal amplification buffer when stored in bulk.	The provided formulation is for 10 × buffer preparation, where certain components may precipitate over time.	Store in small aliquots at room temperature, avoiding repeated freeze-thaw cycles. Vortex thoroughly to disperse precipitate and heat at 65 °C for 10 min or until completely dissolved.
**7**	White colonies selected from an IPTG/X-Gal plate lack the target gene fragment (negative colony PCR).	(1) Colony selection errors due to dense growth on the plate. (2) Non-functional *lacZ* gene preventing β-galactosidase production, leading to white colonies. (3) Unintended DNA integration into *lacZ*, creating false-positive white colonies.	(1) Reduce the volume of competent cells plated to allow better colony separation for easier selection. (2) Chill the plate at 4 °C for 1–2 h before colony picking; some initially white colonies may turn blue. (3) Store plates at 4 °C for a few hours post 37 °C overnight incubation to enhance pigment precipitation, improving color differentiation.
**14**	Premix solution turns orange before amplification.	(1) Incompatible elution buffer—contamination with SDS, EDTA, or acidic compounds. (2) Prolonged exposure to ambient air during preparation.	(1) Dissolve DNA in nuclease-free water only. Avoid TE buffer or elution buffers containing SDS. (2) Minimize exposure by sealing tubes immediately after aliquoting.
**18**	(1) Negative control reaction turns yellow post-incubation. (2) Positive control reaction fails to turn yellow. (3) Positive reaction produces an unclear orange color.	(1) Reagent contamination from positive control or carry-over contamination; excessive incubation beyond 75 min. (2) Poor mixing or degradation of positive control template. (3) Uneven thermal distribution within the heat source. (4) Incomplete color development.	(1) Decontaminate surfaces with 10 % bleach, DNAZap™, or RNase AWAY™. Use barrier tips and limit incubation to 75 min. (2) Ensure thorough mixing of reaction components before incubation and proper reagent storage. (3) Verify temperature consistency within each reaction well using a thermometer. (4) Maintain incubation for at least 60 min at 63 °C—temperature deviations may impair enzyme activity and color development.
**20**	NanoDrop™ spectrophotometer generates an error when measuring colorimetric cLAMP products.	The reaction mixture contains multiple components, including xylenol orange, which absorbs across 300–700 nm, triggering an instrument warning.	Repeat the measurement and confirm the recorded values.

We acknowledge that the clinical sample set used in this study was skewed toward specimens with high viral loads, with most qPCR Ct values below 20 and only one sample reaching a Ct of 32. This distribution reflects the nature of the available samples, which were primarily derived from cutaneous lesion material, known to harbor high viral loads during the mid to late stages of infection. Importantly, the sample archive we had access to contained only one specimen with a Ct value as low as ∼32. We were unable to obtain samples with lower viral loads for further evaluation. In our validation, the cLAMP assay demonstrated 100 % concordance with qPCR results across 50 clinical samples, using a positivity threshold of Ct ≤ 38. The performance is consistent with prior reports, such as Santacruz Tinoco et al. (2025) [39], where median Ct values varied by sample type, pustules and crusts showed Ct values around 23, while pharyngeal exudates reached a median of 34.24. According to CDC guidelines, lesion samples are considered the most reliable for Mpox diagnosis due to their high viral burden [40]. Thus, in clinical contexts where lesion samples are available, our assay is expected to perform with high diagnostic accuracy. However, for non-lesion specimens or borderline Ct values, qPCR confirmation or clinical assessment may be warranted.

## Prospects and limitations

Our proof-of-concept LAMP-XO protocol was formerly described for the detection of *E. coli* [4]. Since then, the assay has served as a steppingstone for various other assays to be developed to address the diagnostic needs in a wide range of emerging point-of-need applications, predominately those in aquaculture [5,10,11]. In this study, we further optimized it to detect Mpox. This optimization not only provides a new affordable (approximately $3/test), and fast (<1.5 h) Mpox screening assay but also reinforces the robustness and adaptability of our cLAMP method, demonstrating its applicability beyond bacterial detection to include virus diagnostics. The successful implementation of this assay highlights its potential as a standardized model, providing a versatile platform for researchers looking to develop cLAMP-based protocols for detecting a wide range of pathogens. By establishing this protocol as a benchmark for future adaptations, our work lays the foundation for extending cLAMP applications to various emerging and re-emerging pathogens, ensuring greater accessibility and reliability in rapid disease detection. As for the limitation, the cLAMP technique is an endpoint assay, which is inherently limited by its lack of quantitative assessment that correlates the cycle threshold with the concentration of the target present in clinical samples. However, if quantitative analysis is needed, performing cLAMP on a Realtime Turbidimeter is required. In addition, reactions must be performed in thermal cyclers or heating blocks with temperature-controlled lids. In cases where lidless heating devices are used, overlaying cLAMP solution with mineral oil prior to incubation is required to prevent evaporation. To comprehensively assess the real-world performance of the test kit, validation should ideally encompass the entire workflow, from sample collection to result readout, using clinical specimens. Alternatively, spiking plasmid DNA into negative clinical samples would have offered a representative measure of clinical sensitivity too. Unfortunately, due to the expiration of our human ethics approval at the time of manuscript revision, we were unable to access Mpox-positive patient specimens to pursue this experiment. Nevertheless, a recent study has demonstrated that extraction-free sample preparation is compatible with LAMP-based diagnostics [41], suggesting that our assay may also tolerate simplified input formats.

In addition, several studies have demonstrated that rapid nucleic acid extraction methods are also compatible with colorimetric isothermal amplification, offering promising alternatives for streamlining the workflow [5,42,43]. Researchers looking to implement this protocol in their laboratories are encouraged to explore suitable rapid sample preparation methods that consistently yield sufficient DNA for analysis.

## CRediT authorship contribution statement

**Wansadaj Jaroenram:** Conceptualization, Data curation, Formal analysis, Funding acquisition, Investigation, Methodology, Project administration, Resources, Supervision, Validation, Visualization, Writing – original draft, Writing – review & editing. **Yada Ongchanchai:** Methodology. **Ravee Nitiyanontakij:** Investigation, Methodology, Formal analysis. **Pawita Suwanwatthana:** Investigation, Methodology, Formal analysis. **Ratchana Ponang:** Investigation, Methodology, Formal analysis. **Wansika Kiatpathomchai:** Funding acquisition.

## Declaration of interests

The authors declare that they have no known competing financial interests or personal relationships that could have appeared to influence the work reported in this paper.

## Data Availability

Data will be made available on request.

## Appendix Supplementary materials

Image, application 1
