# Supplementary material for: Validation and application of a visual LAMP assay for Mpox diagnosis — Insights from clinical samples in Thailand
Source: MethodsX. 2025 Aug 15;15:103568. doi: 10.1016/j.mex.2025.103568 (PMC12446657; doi:10.1016/j.mex.2025.103568)
Supplement: Supplementary file 1 [file mmc1.docx]

**Supplementary information**

**Article title**

Validation and Application of a Visual LAMP Assay for Mpox diagnosis — Insights from Clinical Samples in Thailand

**Authors**

Wansadaj Jaroenram^1,*^, Yada Ongchanchai^1,2^, Ravee Nitiyanontakij^3^, Pawita Suwanwatthana^3^, Ratchana Ponang^3^, Wansika Kiatpathomchai^1^

**Affiliations**

^1^National Center for Genetic Engineering and Biotechnology (BIOTEC), 113 Thailand Science Park, Phahonyothin Rd., Klong Neung, Klong Luang, Pathum Thani 1212

^2^Faculty of Veterinary Technology, Kasetsart University, Bangkok 10900, Thailand

^3^Bamrasnaradura Infectious Diseases Institute, 126 Tivanon Rd., Talat Khwan, Mueang, Nonthaburi 11000, Thailand

**Corresponding author’s email address**

Correspondence: [wansadaj.jar@biotec.or.th](mailto:wansadaj.jar@biotec.or.th)

**Fig. S1.** Genome-level and primer-level alignment analyses evaluating LAMP assay specificity against cowpox virus. (A) Whole-genome alignment between cowpox (NC_003663.2) and Mpox (PV167035.1) reveals high sequence similarity, with 97.28% identity and 89% query coverage, indicating a close genetic relationship. (B) In silico alignment of the cowpox genome with all LAMP primers used in the assay shows no cross-reactivity, supporting primer specificity


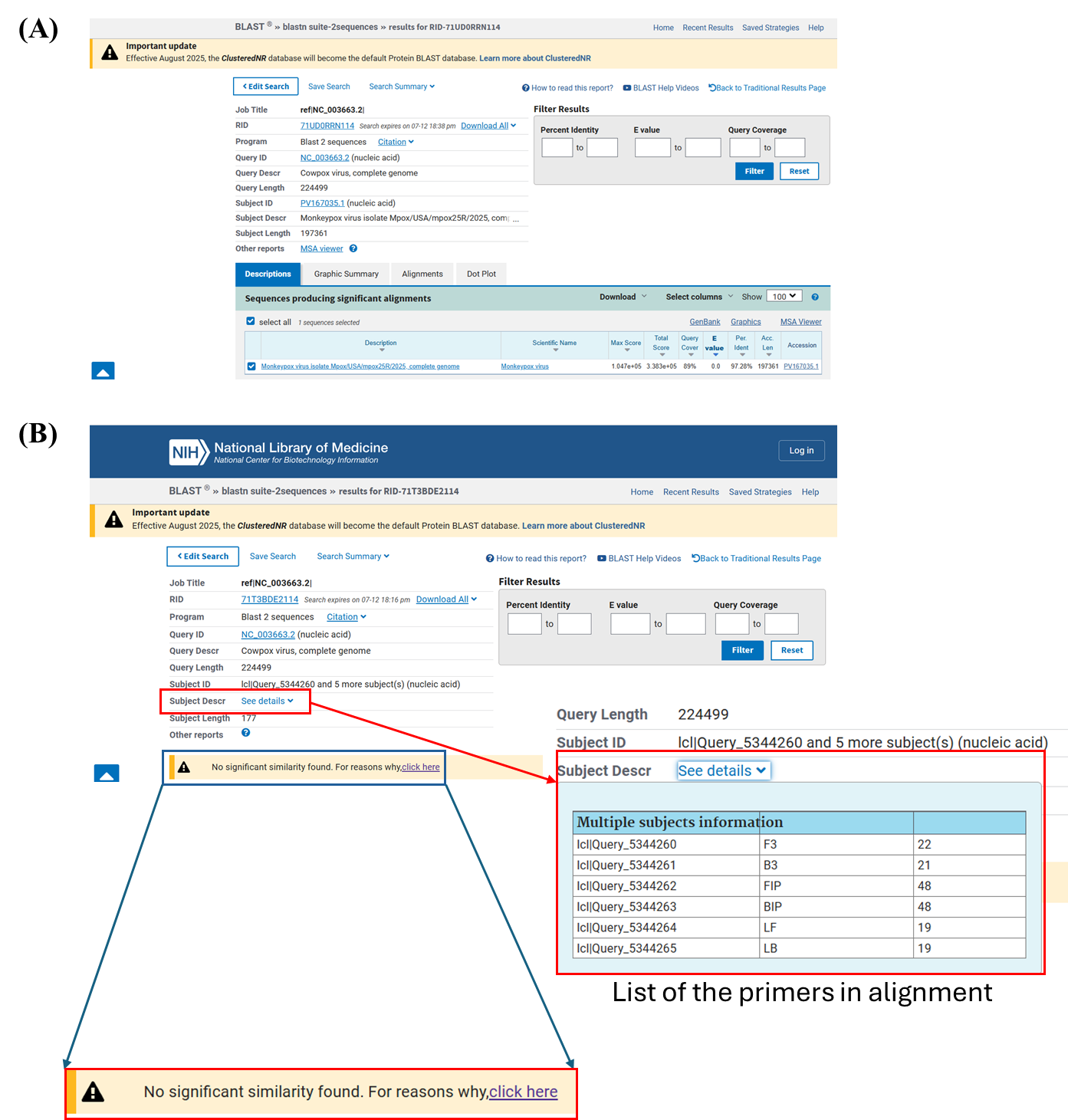


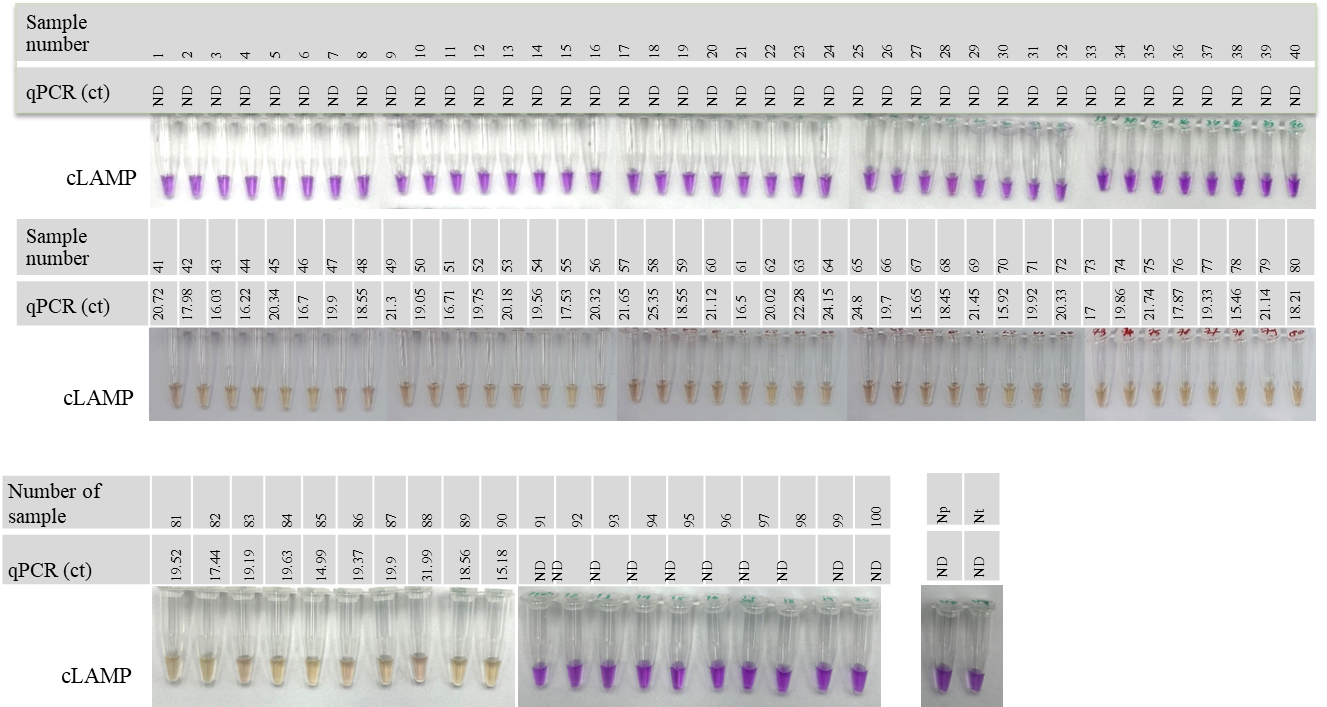


**Fig. S2.** **Proficiency testing of the Mpox-cLAMP assays on clinical samples with known Mpox status determined by qPCR.** Corresponding Ct values of Bioperfectus monkeypox virus real-time PCR kit (BioPerfectus Technologies, Taizhou, China) for the test samples are shown below the tubes. The assay identified 50 Mpox negative (No. 1- 40, 91-100) and all positive correctly (No. 41-90) (n = 100). Np and Nt: template-free control reactions.

**Table S1.** **Components of the cLAMP premix solution.**

| Reagents | Volume (µl/reaction) | Final concentration |
| --- | --- | --- |
| 10 µM F3 | 0.4 | 0.16 uM |
| 10 µM B3 | 0.4 | 0.16 uM |
| 100 µM FIP | 0.4 | 1.6 uM |
| 100 µM BIP | 0.4 | 1.6 uM |
| 10 µM LF | 1.0 | 0.4 uM |
| 10 µM LB | 1.0 | 0.4 uM |
| 10× Low buffer, pH 8.5 | 2.5 | 1X |
| 10 mM dNTPs | 3.5 | 1.4 mM |
| 100 mM MgSO4 | 1.5 | 6 mM |
| 5M Betaine | 2.0 | 0.4 M |
| 8 Units μL^-1^ *Bst* 2.0 WarmStart™ DNA polymerase | 1.0 | 8U |
| DNase-, RNase-free water | 8.65 |  |
| 5 mM XO | 0.25 | 0.05 mM |
| DNA template | 2 |  |

**Table S2.** Types of clinical samples used in the clinical validation stage (Fig. S1).

| **Sample number** | **Results by qPCR** | **Specimen type** |
| --- | --- | --- |
| 1 | Not detected | Blood |
| 2 | Not detected | Skin lesion |
| 3 | Not detected | Oropharyngeal swab |
| 4 | Not detected | Skin lesion |
| 5 | Not detected | Skin lesion |
| 6 | Not detected | Skin lesion |
| 7 | Not detected | Skin lesion |
| 8 | Not detected | Skin lesion |
| 9 | Not detected | Skin lesion |
| 10 | Not detected | Oropharyngeal swab |
| 11 | Not detected | Skin lesion |
| 12 | Not detected | Skin lesion |
| 13 | Not detected | Skin lesion |
| 14 | Not detected | Skin lesion |
| 15 | Not detected | Skin lesion |
| 16 | Not detected | Skin lesion |
| 17 | Not detected | Blood |
| 18 | Not detected | Skin lesion |
| 19 | Not detected | Skin lesion |
| 20 | Not detected | Skin lesion |
| 21 | Not detected | Skin lesion |
| 22 | Not detected | Oropharyngeal swab |
| 23 | Not detected | Skin lesion |
| 24 | Not detected | Skin lesion |
| 25 | Not detected | Rectal swab |
| 26 | Not detected | Oropharyngeal swab |
| 27 | Not detected | Oropharyngeal swab |
| 28 | Not detected | Oropharyngeal swab |
| 29 | Not detected | Blood |
| 30 | Not detected | Skin lesion |
| 31 | Not detected | Skin lesion |
| 32 | Not detected | Skin lesion |
| 33 | Not detected | Skin lesion |
| 34 | Not detected | Skin lesion |
| 35 | Not detected | Skin lesion |
| 36 | Not detected | Skin lesion |
| 37 | Not detected | Oropharyngeal swab |
| 38 | Not detected | Skin lesion |
| 39 | Not detected | Skin lesion |
| 40 | Not detected | Skin lesion |
| 41 | 20.72 | Skin lesion |
| 42 | 17.98 | Skin lesion |
| 43 | 16.03 | Skin lesion |
| 44 | 16.22 | Skin lesion |
| 45 | 20.34 | Skin lesion |
| 46 | 16.7 | Skin lesion |
| 47 | 19.9 | Skin lesion |
| 48 | 18.55 | Skin lesion |
| 49 | 21.3 | Skin lesion |
| 50 | 19.05 | Skin lesion |
| 51 | 16.71 | Skin lesion |
| 52 | 19.75 | Skin lesion |
| 53 | 20.18 | Skin lesion |
| 54 | 19.56 | Skin lesion |
| 55 | 17.53 | Skin lesion |
| 56 | 20.32 | Skin lesion |
| 57 | 21.65 | Skin lesion |
| 58 | 25.35 | Skin lesion |
| 59 | 18.55 | Skin lesion |
| 50 | 21.12 | Skin lesion |
| 61 | 16.5 | Skin lesion |
| 62 | 20.02 | Skin lesion |
| 63 | 22.28 | Skin lesion |
| 64 | 24.15 | Skin lesion |
| 65 | 24.8 | Skin lesion |
| 66 | 19.7 | Skin lesion |
| 67 | 15.65 | Skin lesion |
| 68 | 18.45 | Skin lesion |
| 69 | 21.45 | Skin lesion |
| 70 | 15.92 | Skin lesion |
| 71 | 19.92 | Skin lesion |
| 72 | 20.33 | Skin lesion |
| 73 | 17 | Skin lesion |
| 74 | 19.86 | Skin lesion |
| 75 | 21.74 | Skin lesion |
| 76 | 17.87 | Skin lesion |
| 77 | 19.33 | Skin lesion |
| 78 | 15.46 | Skin lesion |
| 79 | 21.14 | Skin lesion |
| 80 | 18.21 | Skin lesion |
| 81 | 19.52 | Skin lesion |
| 82 | 17.44 | Skin lesion |
| 83 | 19.19 | Skin lesion |
| 84 | 19.63 | Skin lesion |
| 85 | 14.99 | Skin lesion |
| 86 | 19.37 | Skin lesion |
| 87 | 19.9 | Skin lesion |
| 88 | 31.99 | Skin lesion |
| 89 | 18.56 | Skin lesion |
| 90 | 15.18 | Skin lesion |
| 91 | Not detected | Skin lesion |
| 92 | Not detected | Oropharyngeal swab |
| 93 | Not detected | Oropharyngeal swab |
| 94 | Not detected | Oropharyngeal swab |
| 95 | Not detected | Oropharyngeal swab |
| 96 | Not detected | Oropharyngeal swab |
| 97 | Not detected | Oropharyngeal swab |
| 98 | Not detected | Oropharyngeal swab |
| 99 | Not detected | Blood |
| 100 | Not detected | Skin lesion |
